# Antitumor Phenylpropanoids Found in Essential Oils

**DOI:** 10.1155/2015/392674

**Published:** 2015-01-15

**Authors:** Adriana Andrade Carvalho, Luciana Nalone Andrade, Élida Batista Vieira de Sousa, Damião Pergentino de Sousa

**Affiliations:** ^1^Núcleo de Farmácia, Universidade Federal de Sergipe, 58051-970 Lagarto, SE, Brazil; ^2^Departamento de Farmácia, Universidade Federal de Sergipe, 49100-000 São Cristóvão, SE, Brazil; ^3^Departamento de Ciências Farmacêuticas, Universidade Federal da Paraíba, CP 5009, 58051-970 João Pessoa, PB, Brazil

## Abstract

The search for new bioactive substances with anticancer activity and the understanding of their mechanisms of action are high-priorities in the research effort toward more effective treatments for cancer. The phenylpropanoids are natural products found in many aromatic and medicinal plants, food, and essential oils. They exhibit various pharmacological activities and have applications in the pharmaceutical industry. In this review, the anticancer potential of 17 phenylpropanoids and derivatives from essential oils is discussed. Chemical structures, experimental report, and mechanisms of action of bioactive substances are presented.

## 1. Introduction

Cancer is a global health concern that causes mortality in both children and adults. More than 100 distinct types and subtypes of cancer can be found within specific organs [1]. Despite the success of several cancer therapies, an ideal anticancer drug has not been discovered, and numerous side effects limit treatment. However, research into new drugs has revealed a variety of new chemical structures and potent biological activities that are of interest in the context of cancer treatment.

Essential oils are natural products that are a mixture of volatile lipophilic substances. The chemical composition of essential oils includes monoterpenes, sesquiterpenes, and phenylpropanoids, which are usually oxidized in an aliphatic chain or aromatic ring. Several studies have shown that this chemical class has several biological activities, including analgesic, anticonvulsant, and anti-inflammatory effects [2–4]. Antitumor activity has been reported for essential oils against several tumor cell lines [5–7], and these oils contain a high percentage of phenylpropanoids, which are believed to contribute to their pharmacological activity [8, 9].

This paper presents a literature review of phenylpropanoids from essential oils with respect to antitumor activity, with chemical structures and names of bioactive compounds provided. The phenylpropanoids presented in this review were selected on the basis of effects shown in specific experimental models for evaluation of antitumor activity and/or by complementary studies aimed at elucidating mechanisms of action (Table 1). The selection of essential oil constituents in the database was related to various terms, including essential oils and phenylpropanoids, as well as names of representative compounds of chemical groups, and refined with respect to antitumor activity, cytotoxic activity, and cytotoxicity. The search was performed using scientific literature databases and Chemical Abstracts Service (CAS) in November 2013.

## 2. Phenylpropanoids

### 2.1. Eugenol

Eugenol is the active component of essential oil isolated from clove (*Syzygium aromaticum*) and has antimutagenic, antigenotoxic, and anti-inflammatory properties [10]. Eugenol also has cytotoxic activity. This drugs can induce cell death in several tumor and cell types: mast cells [11–13], breast adenocarcinoma [13], melanoma cells [14–16], leukemia [14, 17], colon carcinoma [18], cervical carcinoma [19–23], prostate cancer [24], submandibular gland adenocarcinoma [25], human dental pulp [26], murine peritoneal macrophages [27], androgen-insensitive prostate cancer, oral squamous carcinoma [17, 28], human submandibular gland carcinoma [29, 30], salivary gland [30], gingival fibroblasts [31–33], hepatoma [34], human dental pulp cells [35], human gingival fibroblasts, and epidermoid carcinoma cells derived from human submandibular gland [36, 37]. Furthermore, eugenol is neither carcinogenic nor mutagenic and is not cytotoxic in lymphocytes [14]. Isoeugenol was found to be more toxic than eugenol when the cytotoxicity of isoeugenol, bis-eugenol, and eugenol was tested in HSG (human submandibular gland adenocarcinoma) cell lines [25]. In this way, Atsumi and collaborators [37] compared the cytotoxicity of dehydrodiisoeugenol, alpha-di-isoeugenol, isoeugenol, eugenol, and bis-eugenol in a gland tumor cell line (HSG) and normal human gingival fibroblasts (HGF). Both the cytotoxic activity and the DNA synthesis inhibitory activity of these compounds against the salivary gland tumor cell line (HSG) and normal human gingival fibroblasts (HGF) were greatest in dehydrodiisoeugenol and alpha-di-isoeugenol, followed by isoeugenol, which showed greater activity than eugenol [37].

Synergistic effects have been demonstrated for eugenol with gemcitabine and fluorouracil, which potentiated its cytotoxic effect on HeLa cells (human cervical carcinoma) [19, 20, 38]. Eugenol also significantly decreased expression of Bcl-2, COX2, and IL-1b in the HeLa cell line [20]. Atsumi and collaborators [39] demonstrated that the effects of eugenol on ROS production were biphasic, with production enhanced at lower eugenol concentrations (5–10 *μ*M) and inhibited at higher concentrations (500 *μ*M). Suzuki and collaborators [40] demonstrated that eugenol stimulated production of superoxide (O_2_^−^) free radicals in guinea pig neutrophils without lag time.

Eugenol halts cells in the replication phase, suggesting that cells stop to repair DNA damage and either reenter the cell cycle or, in cases of massive DNA damage, activate apoptosis. Melanoma cells treated with eugenol remain in the S phase and undergo apoptosis, and eugenol treatment upregulates numerous enzymes involved in the base excision repair pathway, including E2F family members [15].

In another study, eugenol at higher doses induced chromosomal aberrations, with significant increases (3.5%) in aberrant cells at a concentration of 2500 *μ*M in V79 cells (Chinese hamster lung fibroblast). Eugenol was also assayed for genotoxic activity via inhibition of topoisomerase II and showed dose-dependent inhibition [41].

The chemopreventive potential of eugenol was also studied [10]. Using* in vivo* methods, Pal and collaborators [10] showed that eugenol inhibits skin carcinogenesis induced by dimethylbenz[a]anthracene (DMBA) croton oil in mice, probably due to inhibition of proliferation-associated genes (c-Myc and H-ras) and antiapoptotic gene Bcl2, along with upregulation of proapoptotic genes Bax, p53, and active caspase-3 [10]. Kaur and collaborators [42] studied the chemopreventive effect of eugenol in DMBA/TPA-induced carcinogenesis in murine skin. They showed that topical application of eugenol resulted in a marked decline in hyperplasia, epidermal ODC activity, protein expression of iNOS and COX-2, and secretion of proinflammatory cytokines, all of which are classical markers of inflammation and tumor promotion [42]. In addition, eugenol has been shown to produce antioxidant effects via free radical scavenging activity and reduction of ROS [22, 36, 43]. Atsumi and collaborators [36] showed that visible-light irradiation and elevation of the pH of the eugenol-containing medium resulted in significantly lower cell survival in HSG cultures in comparison with eugenol alone.

*In vivo* murine assays have also demonstrated the antitumor potential of eugenol. Treatment of female B6D2F1 mice bearing B16 melanoma allografts with 125 mg/kg of eugenol resulted in a small, but highly significant (*P* = 0.0057), 2.4-day tumor growth delay. Furthermore, the treated animals had no fatalities that were attributed to metastasis or tumor invasion, which is indicative of the ability of eugenol to suppress melanoma metastasis [15]. Jaganathan and collaborators [44] also demonstrated the antitumor potential of eugenol using an* in vivo* assay, in which a dose of 100 mg/kg caused 24.35% tumor growth inhibition and inhibited the growth of Ehrlich ascites by 28.88%. In contrast, Tangke Arung and collaborators [45] showed that 100 *μ*g/mL eugenol inhibited melanin formation by more than 42% in the B16 melanoma cell line* in vitro*, with cytotoxicity in 5% of cells. At a higher concentration of 200 *μ*g/mL 23% cytotoxicity was observed, which demonstrated that eugenol could be useful as a skin-whitening agent for the treatment of hyperpigmentation [45].

Furthermore, it has been demonstrated that eugenol, when mixed with zinc oxide, has a restorative effect on dental erosion and demineralization [46]. Using human dental pulp cells (D824) it was observed that eugenol had a cytotoxic effect, with reduction of cell growth and inhibition of colony-forming cell [35]. D824 cells have the potential for metabolic activation, because they are a mixed-cell population composed of many types of cells, and thus the cytotoxic activity of eugenol could be attributable to eugenol metabolites. However, Marya and collaborators [46] showed a hemolytic effect of eugenol, which could be a possible side effect of this drug. In addition, Anpo and collaborators [35] showed that eugenol reduced growth and survival of human dental pulp cells, as well as collagen synthesis and bone sialoprotein (BSP) expression, which play a critical role in physiological and reparative dentinogenesis. Eugenol is a phenylpropanoid with promising antitumor drug profile. Further studies to elucidate the mechanisms that mediate the adverse effects of eugenol are necessary.

### 2.2. Methyleugenol, Isoeugenol, Methylisoeugenol, and 1′-Hydroxymethyleugenol

Methyleugenol is a substituted alkenylbenzene found in a variety of foods and essential oils. It is structurally similar to eugenol and found in many plant species [47]. Methyleugenol produced cytotoxic effects in rat and mouse hepatocytes [47, 48] and leukemia [48]. Methyleugenol also produced genotoxicity in mice [47] and in cultured cells [49] and caused neoplastic lesions in the livers of Fischer 344 rats and B6C3F1 mice [47].

Isoeugenol is a phenylpropanoid produced by plants. As a flavoring agent, isoeugenol is added to nonalcoholic drinks, baked foods, and chewing gums. In male F344/N rats, isoeugenol showed carcinogenic effects, causing increased incidence of rarely occurring thymoma and mammary gland carcinoma. There was no evidence of carcinogenic activity due to isoeugenol in female F344/N rats. However, there was clear evidence of carcinogenic activity due to isoeugenol in male B6C3F1 mice, including increased incidence of hepatocellular adenoma, hepatocellular carcinoma, and hepatocellular adenoma with carcinoma. Carcinogenic activity due to isoeugenol in female B6C3F1 mice was observed in the form of increased incidence of histiocytic sarcoma. Exposure to isoeugenol resulted in nonneoplastic lesions of the nose in male and female rats, of the kidney in female mice, and of the nose, forestomach, and glandular stomach in mice of both sexes [50]. However, methyleugenol is minimally cytotoxic for hepatocytes and leukemia cells compared to eugenol [48, 49]. The structural similarity of these substances with eugenol stimulates advances in pharmacological studies to explore their therapeutic potential in cancer treatment.

### 2.3. Safrole, Safrole-2′,3′-oxide, and Myristicin

Safrole is an important food-borne phytotoxin found in many natural products, such as oil of sassafras, anise, basil, nutmeg, and pepper. Safrole is cytotoxic against human tongue squamous carcinoma [51], primary human buccal mucosal fibroblasts [52], prostate cancer [53], rat hepatocytes [54], and leukemia [51] and shows genotoxic activity [55, 56].

Safrole induced apoptosis in human tongue squamous carcinoma SCC-4 cells by mitochondria- and caspase-dependent signaling pathways. Safrole-induced apoptosis was accompanied by upregulation of Bax and Bid and downregulation of Bcl-2, which increased the ratio of Bax/Bcl-2, resulting in cytochrome c release, increased Apaf-1 levels, and sequential activation of caspase-9 and caspase-3 in a time-dependent manner [51]. In A549 human lung cancer cells, safrole activated caspases 3, 8, and 9 [57]. In rat hepatocytes cells, safrole induced cell death by loss of mitochondrial membrane potential and generation of oxygen radical species, which were assayed using 2′,7′-dichlorodihydrofluorescein diacetate (DCFH-DA) [54].

Fan and collaborators [58] showed that safrole promoted the activities of macrophages and NK cells in BALB/c mice. While promoting macrophage phagocytosis, safrole increased abundance of cell markers such as CD11b and Mac-3. Additionally, NK cell cytotoxicity was remarkably suppressed in mice treated with safrole, as were levels of cell markers for T cells (CD3) and B cells (CD19). Safrole was also cytotoxic against primary human buccal mucosal fibroblasts (BMFs) [52]. Ni and collaborators [52] demonstrated that safrole increased NF-*κ*B expression, which may have been involved in the pathogenesis of oral submucous fibrosis. NF-*κ*B expression induced by safrole in fibroblasts may be mediated by ERK activation and the COX-2 signal transduction pathway.

A study by Chang and collaborators [53] investigated the effect of safrole on intracellular Ca^2+^ mobilization and viability of human PC3 prostate cancer cells. Cytosolic free Ca^2+^ levels ([Ca^2+^]_*i*_) were measured using fura-2 as a probe. Safrole increased [Ca^2+^]_*i*_ by causing Ca^2+^ release from the endoplasmic reticulum in a phospholipase C- and protein kinase C-independent manner, which decreased cell viability in a concentration-dependent manner. In HL-60 leukemia cells, safrole promoted the expression of glucose-regulated protein 78 (GRP78), growth arrest- and DNA damage-inducible gene 153 (GADD153), and activating transcription factor 6*α* (ATF-6*α*) [51]. In the unscheduled DNA synthesis (UDS) assay described by Howes and collaborators [55], safrole exhibited genotoxic activity in freshly isolated rat hepatocyte primary cultures.

Safrole-2′,3′-oxide (SAFO) is a reactive electrophilic metabolite of safrole. SAFO is the most mutagenic metabolite of safrole that has been tested in the Ames test, but data on the genotoxicity of SAFO in mammalian systems is scarce. SAFO induced cytotoxicity, DNA strand breakage, and micronuclei formation in human cells* in vitro* and in mice [56]. In addition, safrole produced mutagenicity in* Salmonella* TA 98 and TA 100 in the Ames test [59].

Myristicin (1-allyl-3,4-methylenedioxy-5-methoxybenzene) is an active constituent of nutmeg, parsley, and carrot. A study by Lee and collaborators [60] investigated the cytotoxic and apoptotic effects of myristicin on human neuroblastoma SK-N-SH cells. Apoptosis triggered by myristicin was caused by cleavage of PARP, which was accompanied by accumulation of cytochrome c and activation of caspase-3. These results suggested that myristicin induced cytotoxicity in human neuroblastoma SK-N-SH cells by an apoptotic mechanism [60].

Ahmad and collaborators [61] investigated the effect of myristicin on activity of glutathione S-transferase (GST) and NADPH:quinone oxidoreductase (QR) in four mouse strains. The authors showed that activity of GST and QR was significantly increased in the livers of all four mouse strains, GST activity was increased in the intestine of three out of four strains, and QR activity was significantly increased in the lungs and stomachs of three out of four stains. Thus myristicin, which is found in a wide variety of herbs and vegetables, shows strong potential as an effective chemoprotective agent against cancer.

Safrole, safrole-2′,3′-oxide, and myristicin are bioactive substances in antitumor models that can be used as starting materials for the preparation of derivatives with improved pharmacological profile.

### 2.4. Estragole, Anethole, and trans-Anethole Oxide

Estragole has been isolated from essential oils of* Artemisia dracunculus* and* Leonotis ocymifolia*. Howes and collaborators [55] demonstrated the genotoxic activity of estragole via UDS assay, in which estragole induced dose-dependent increases in UDS up to 2.7 times that of the control in rat hepatocytes in primary culture.

Anethole (1-methoxy-4-(1-propenyl)benzene) occurs naturally as a major component of essential oils from fennel and star anise and is also present in numerous plants such as dill, basil, and tarragon [62]. Anethole had a cytotoxic effect on fibrosarcoma tumor [63], breast cancer [63], hepatocytes [55, 64], cervical carcinoma [21, 23], and Ehrlich ascites tumor [65], as well as an anticarcinogenic effect and a lack of clastogenic potential [65].

Chainy and collaborators [66] reported that anethole reduced apoptosis by inhibiting induction of NF-*κ*B, activator protein 1 (AP-1), c-jun N-terminal kinase (JNK), and mitogen-activated protein kinase kinase (MAPKK) by tumor necrosis factor (TNF). Choo and collaborators investigated the antimetastatic activity of anethole [63] and showed that anethole inhibited proliferation, adhesion, and invasion of highly metastatic human HT-1080 fibrosarcoma cells. Anethole also inhibited the activity of metalloproteinases (MMP-2 and MMP-9) and increased the activity of MMP inhibitor TIMP-1 [63]. Nakagawa and Suzuki [62] showed that anethole induced a concentration- and time-dependent loss of cell viability in isolated rat hepatocytes, which was followed by decreases in intracellular levels of ATP and total adenine nucleotide pools. Howes and collaborators [55] demonstrated that anethole did not induce unscheduled DNA synthesis (UDS) in rat hepatocytes in primary culture. In Ehrlich ascites tumor-bearing mice,anethole increased survival time and reduced tumor weight, tumor volume, and body weight [65].

Anethole is metabolized through 3 pathways:* O*-demethylation, *ω*-hydroxylation followed by side chain oxidation, and epoxidation of the 1,2-double bond. The cytotoxicity of* trans*-anethole oxide in rat hepatocytes has been shown to be due to its metabolism to epoxide [67]. In addition,* trans*-anethole oxide produced a positive result in the* Salmonella* mutation assay and induced tumors in mice. These results suggest that epoxidation of the side chain of anethole* in vivo* could be a carcinogenic metabolic mechanism. Kim and collaborators [67] found that* trans*-anethole oxide is more toxic to animals than* trans*-anethole and was mutagenic in point mutation and frameshift mutation Ames test models.* trans*-Anethole did not induce hepatomas in male B6C3F1 mice, but the highest dose of* trans*-anethole oxide tested (0.5 *μ*mol/g) significantly increased the incidence of hepatomas.

### 2.5. Asaraldehyde, *β*-Asarone, and trans-Asarone Oxide

*Acorus gramineus* (Araceae), which is distributed throughout Korea, Japan, and China, has been used in Korean traditional medicine for improvement of learning and memory, sedation, and analgesia [68]. Several pharmacologically active compounds, such as *β*-asarone, *α*-asarone, and phenylpropenes, have been reported from this rhizome [69]. Park and collaborators [70] investigated asarone and asaraldehyde and showed minimal cytotoxicity (IC_50_ < 30 *μ*M) in the SRB assay using 4 human tumor cell lines: A549 (non-small cell lung adenocarcinoma), SK-OV-3 (ovarian cancer cell), SK-MEL-2 (skin melanoma), and HCT15 (colon cancer cell).* trans*-Asarone oxide, prepared from* trans*-asarone and dimethyldioxirane, induced hepatomas in B6C3F1 mice and skin papillomas in CD-1 mice and was mutagenic for* Salmonella* strains [67].

### 2.6. Cinnamaldehyde, 2′-Hydroxycinnamaldehyde, and Cinnamic Acid

Cinnamaldehyde is a bioactive compound isolated from the stem bark of* Cinnamomum cassia* and has been widely used in folk medicine for its anticancer [71], antibacterial [72], antimutagenic [73], and immunomodulatory effects [74], as well as to remedy other diseases [75]. The cytotoxic activity of cinnamaldehyde has been confirmed in melanoma [76, 77], the colon [76, 78, 79], breast cancer [78], hepatic tumor [80, 81], leukemia [71, 82, 83], cervical carcinoma [76, 83] the lung, the ovary, the central nervous system [76], lymphoma, mouse leukemia [76, 84], mouse lung carcinoma [71], lymphocytes [74], hepatocytes [85], embryo cells [86], and larynx carcinoma [87]. Its genotoxicity has been confirmed* in vitro* [87]. Cinnamaldehyde also had genotoxic effects against SA7-transformed Syrian hamster embryo cells [86].

Ng and Wu [80] showed that cinnamaldehyde induced lipid peroxidation in hepatocytes isolated from male Sprague-Dawley rats with glutathione depletion. Adding NADH generators, for example, xylitol, prevented cytotoxicity induced by cinnamaldehyde, but decreasing mitochondrial NAD^+^ with rotenone markedly increased cinnamaldehyde cytotoxicity. The authors showed that cinnamaldehyde-induced cytotoxicity and inhibition of mitochondrial respiration were markedly increased by ALDH inhibitors and in particular by cyanamide [80].

Chew and collaborators [78] used flow cytometric analysis to show that 80 *μ*M of cinnamaldehyde caused cell cycle arrest at the G_2_/M phase in HCT 116 cells and induced cleavage of caspase-3 and PARP. It has also been proposed that cinnamaldehyde induced apoptosis by ROS release with TrxR-inhibitory and Nrf2-inducing properties [78]. Ka and collaborators [71] demonstrated that cinnamaldehyde induced ROS-mediated mitochondrial permeability and cytochrome c release in human leukemia cells (HL-60).

Using hepatoma cells, Wu and collaborators [81] demonstrated that cinnamaldehyde upregulated Bax protein, downregulated Bcl-2 and Mcl-1, and caused Bid to cleave upon the activation of caspase-8. These events consequently led to cell death. JNK, p38, and ERK were activated and phosphorylated after cinnamaldehyde treatment in a time-dependent manner, which suggested that apoptosis was mediated by activation of proapoptotic Bcl-2 family (Bax and Bid) proteins and MAPK pathways [81]. Cinnamaldehyde can also activate TRPA1 expression in melanoma cells [77].

Cinnamaldehyde caused a time-dependent increase in CD95 (APO-1/CD95) protein expression in HepG2 cells (human hepatoma), while also downregulating antiapoptotic proteins (Bcl-XL) and upregulating proapoptotic (Bax) proteins in a time-dependent manner [80]. Preincubation of HepG2 cells with cinnamaldehyde effectively inhibited the expression of Bax, p53, and CD95, as well as the cleavage of PARP. This pretreatment also prevented downregulation of Bcl-XL [80]. Using the HepG2 and Hep3B human hepatoma cancer cell lines, Chuang and colleagues [88] demonstrated that cinnamaldehyde had a potent inhibitory effect against human hepatoma cell growth. They observed that the JAK2-STAT3/STAT5 pathway might be an important target of cinnamaldehyde. Cinnamaldehyde also altered apoptotic signaling. Cinnamaldehyde significantly decreased protein levels of cyclin D1 and proliferative cell nuclear antigen (PCNA) but increased the protein levels of p27^Kip1^ and p21^Waf1/Cip1^ [86]. In an assay of thioredoxin reductase (TrxR) action, cinnamaldehyde showed a TrxR inactivation effect that could contribute to its cytotoxicity [89]. Furthermore, cinnamaldehyde had an antitumor effect in Sarcoma 180-bearing BALB/c mice and a protective effect on immune function [89].

2′-Hydroxycinnamaldehyde, a cinnamaldehyde derivative, was studied for its immunomodulatory effects. The chemopreventive effects of cinnamaldehyde derivatives were demonstrated on hepatocellular carcinoma formation in H-ras12V transgenic mice, where they probably produced a long-term immunostimulating effect on T cells, because immune cell infiltration into hepatic tissues was increased [90].

2′-Hydroxycinnamaldehyde has immunomodulatory effects* in vivo*, but* in vitro* studies showed that secreted IgM level was depressed in the culture supernatants of splenocytes. Decreased IgM produced by cinnamaldehyde treatment* in vitro *appeared to be due to lower levels of B-cell proliferation, rather than direct inhibition of IgM production [74]. Koh and collaborators [74] also demonstrated that cinnamaldehyde induced T-cell differentiation from CD4CD8 double positive cells to CD4 or CD8 single positive cells.

Cinnamic acid occurs throughout the plant kingdom and particularly in flavor compositions and products containing cinnamon oil [91]. Cinnamic acid inhibited proliferation of uterocervical carcinoma [92], leukemia [93], colon adenocarcinoma [79], glioblastoma, melanoma, prostate, lung carcinoma [94], osteogenic sarcoma [95] cells, Mac Coy cells [96], Hep G2 cells [97], and kidney epithelial (VERO) cells [98].

Cinnamic acid had an inhibitory effect on uterocervical carcinoma (U14) cells in mice, causing tumor cell apoptosis [92].* In vitro* assay of U14 cells demonstrated a shortened G_2_-M period, lengthened cell cycle, and inhibited cell proliferation, which supported the conclusion that cinnamic acid influenced tumor cell cycle [92].

Ekmekcioglu and collaborators [79] showed that cinnamic acid inhibited proliferation and DNA synthesis of Caco-2 (human colon) cells. Treatment with cinnamic acid modulated the Caco-2 cell phenotype by dose-dependently stimulating sucrase and aminopeptidase N activity, while inhibiting alkaline phosphatase activity. In melanoma cells cinnamic acid induced cell differentiation with morphological changes and increased melanin production. Cinnamic acid reduced the invasive capacity of melanoma cells and modulated expression of genes implicated in tumor metastasis (collagenase type IV and tissue inhibitor metalloproteinase 2) and immunogenicity (HLA-A3, class-I major histocompatibility antigen) [94].

Using* in vivo* and* in vitro* assays, Zhang and collaborators (2010) [92] showed that cinnamic acid influenced the cell cycle of uterocervical carcinoma cells (U14); the G_2_-M period was shortened, cell cycle was lengthened, and cell proliferation was inhibited. Cinnamic acid also induced differentiation of human osteogenic sarcoma cells and caused a higher percentage of cells in S phase [95].

### 2.7. Hydroxychavicol and 1′-Acetoxychavicol Acetate

Hydroxychavicol (1-allyl-3,4-dihydroxybenzene) is a major component in* Piper betle* leaf, which is used for betel quid chewing in Asia, and is also a major metabolite of safrole, which is the main component of sassafras oil, in rats and humans. A study by Nakagawa and collaborators [54] demonstrated the biotransformation and cytotoxic effects of hydroxychavicol in freshly-isolated rat hepatocytes. In hepatocytes pretreated with diethyl maleate or salicylamide, hydroxychavicol-induced cytotoxicity was enhanced and was accompanied by a decrease in the formation of conjugates and inhibition of hydroxychavicol loss.

Other studies indicate that mitochondria are the target organelles for hydroxychavicol, which induces cytotoxicity through mitochondrial failure related to mitochondrial membrane potential at an early stage, and lipid peroxidation through oxidative stress at a later stage. Furthermore, the onset of cytotoxicity depends on the initial and residual concentrations of hydroxychavicol, rather than its metabolites.

**Figure 1:**
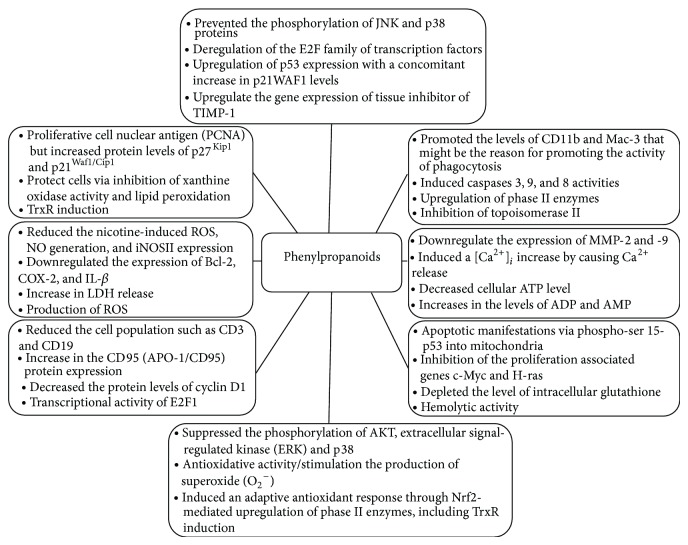
Possible mechanisms of action from phenylpropanoids antitumoral activity.

1′-Acetoxychavicol acetate is obtained from the rhizomes of* Languas galanga* (Zingiberaceae), a traditional condiment in Thailand. Recent studies have revealed that 1′-acetoxychavicol acetate has potent chemopreventive effects against rat oral carcinomas and inhibits chemically induced tumor formation and cellular growth of cancer cells. 1′-Acetoxychavicol acetate inhibited NF-*κ*B and induced apoptosis of myeloma cells* in vitro* and* in vivo*. Therefore, 1′-acetoxychavicol acetate is a novel NF-*κ*B inhibitor and represents a new therapy for the treatment of multiple myeloma patients [99]. The isolation and identification of 1′-acetoxychavicol acetate, an inhibitor of xanthine oxidase, may induce antitumor activity by inhibiting generation of anions during tumor promotion [100] (Figure 1).

## 3. Conclusions

The studies presented in this review reveal the anticancer therapeutic potential of bioactive constituents found in essential oils and medicinal plants, the phenylpropanoids. The research on the clinical studies of these natural products is required to the development of new drug candidates with applications in the therapy of cancer.

## Figures and Tables

**Table 1:** Essential oil phenylpropanoids with antitumoral activity.

Compound	Experimental protocol	Antitumoral activity and/or mechanism	Animal/cell line tested	Reference
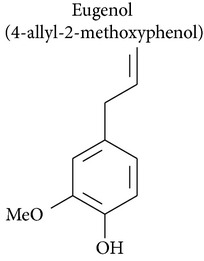	Anaphylaxis model	Apoptotic manifestations via phospho-ser 15-p53 into mitochondria	Mast cells	[11]
	Skin carcinogenesis model	Inhibition of the proliferation associated genes c-Myc and H-ras and antiapoptotic gene Bcl2 along with upregulation of proapoptotic genes Bax, p53, and active caspase-3	Mice	[12]
	Trypan-blue assays	Cytotoxic activity	B16-F10, Sbcl2, WM3211, WM98-1 and WM1205Lu, PC-3, human gingival fibroblasts, oral mucosal, neutrophils—male guinea pig, rat hepatocytes cells	[14, 15, 23, 32, 33, 48, 49]
	Melanoma cell proliferation	Deregulation of the E2F family of transcription factors, transcriptional activity of E2F1	Sbcl2, WM3211, WM98-1, and WM1205Lu cells	[15]
	Flow cytometry analysis	Cytotoxic activity	P-815, K-562, CEM, and MCF-7 cells	[13]
	VL irradiation time	Antioxidative reactivity	HSG, HSC-2, and HL-60 cells	[17]
	MTT assay	Cytotoxic activity	B16-F10, P-815, K-562, CEM, MCF-7, MCF-7 gem, HeLa, DU-145, KB, HSG, human dental pulp, murine peritoneal macrophages HL-60, HepG-2, B16, cells	[13, 19–22, 25–29, 38, 45, 46, 48]
	DPPH assay	Antioxidative activity	Caco-2 cells and VH10 fibroblasts	[18]
	Flow cytometer analysis	Enhanced the accumulation of cells in the S and G2/M phase which may be unable to divide	HeLa cells
	DAPI staining	Increase in the number of apoptotic cells	
	*In vitro* hemolytic activity	Hemolytic activity	Human erythrocytes	[19]
	Caspase-3 colorimetric assay	Induce caspase 3-mediated apoptosis
	RT-PCR	Anticancer activities via apoptosis induction and anti-inflammatory downregulation of Bcl-2, COX-2, and IL-1*β*
	RT-PCR	Downregulated the expression of Bcl-2, COX-2, and IL-*β*	HeLa cells	[20]
	Flow cytometer analysis	Increased population of cells G2/M phase by 4.5-fold	PC-3 cells	[24]
	Western blot and RT-PCR analysis	Reduced expression of antiapoptotic protein Bcl-2 and enhanced expression of proapoptotic protein Bax		
	DPPH radical-scavenging activity	Formation of dimers	HSG cells	[25]
	ELISA	Reduced the nicotine-induced ROS, NO generation, and iNOSII expression	Murine peritoneal macrophages	[27]
	Spectrophotometric analysis	Increase in LDH release	DU-145 and KB cells	[28]
	ESR analysis	Activity of the production of phenoxyl radicals with most efficiently scavenged reactive oxygen	HSG cells	[29]
	Laser cytometry analysis	Production of ROS induced by VL-irradiated is significantly affected by pH		
	Antioxidants production	Produced antioxidants in alkaline solutions	Human salivary gland and oral squamous cells	[30]
	DPPH assay	Apoptosis-inducing effect	HGF and HSG cells	[31]
	TBA analysis lipid oxidation	Depleted intracellular glutathione; protect cells from the genetic attack of reactive oxygen species via inhibition of xanthine oxidase activity and lipid peroxidation	Oral mucosal fibroblasts	[32]
	ATP assay	Decreased cellular ATP level in a concentration- and time-dependent manner		
	NR assay	Intracellular glutathione levels	HFF and HepG2 cells	[33]
	Dichlorofluorescein assay	Reduction in the intracellular level of GSH	HSG cells	[34]
	CAs assay	Induced a dose-dependent increase of aberrant cells	V79 cells	[41]
	Topo II activity assay	Inhibition of topoisomerase II		
	Croton oil induced skin carcinogenesis	Inhibition of the proliferation associated genes c-Myc and H-ras and antiapoptotic gene Bcl2 along with upregulation of proapoptotic genes Bax, p53, and active caspase-3	Swiss mice	[36]
	DMBA/TPA-induced carcinogenesis in murine skin	Declined of hyperplasia, epidermal ODC activity, and protein expression of iNOS, COX-2, and secretion of proinflammatory cytokines	Swiss mice	[42]
	TUNEL assay	Upregulation of p53 expression with a concomitant increase in p21WAF1 levels in epidermal cells indicating induction of damage to the DNA		
	Flow cytometric analysis	cDNA array analysis showed that eugenol caused deregulation of the E2F family of transcription factors	WM1205Lu cells	[24]
	TUNEL assay	Induces apoptosis in melanoma tumors	WM1205Lu cells	
	DPPH assay	Antioxidative properties	HL-60 and HepG-2 cells	[48]
	Sulforhodamine B assay	Cytotoxic activity	SK-OV-3, XF-498, and HCT-15 cells	[76]
	Murine Ehrlich ascites and solid carcinoma models	Inhibit the growth of Ehrlich ascites	BALB/c mice	[44]
	DPPH assay	Antioxidation activity	HepG2 cells	[22]
	Western blot analysis	Decreased the protein expression of BSP in a concentration-dependent manner	Human dental pulp cells	[35]
	DPPH assay	Antioxidant effect	Raw 264.7 cells	[43]
	VL irradiation/MTT assay	Generation of eugenol radicals	HSG and HGF cells	[36]
	Laser cytometer	Generation of ROS		
	ESR analysis	Produced phenoxyl radicals	HSG and HGF cells	[37]
	Superoxide generation/spectrophotometer	Stimulation the production of superoxide (O_2_^−^)	Neutrophils—male guinea pig	[40]

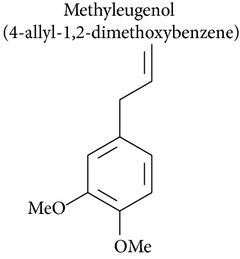	DPPH assay	Antioxidative properties	HL-60 and HepG-2 cells	[48]
	UDS assay	Cytotoxicity and genotoxicity effects	B6C3F1 mouse hepatocytes	[47]
			F-344 rat hepatocytes	
	L-Lactate assay	Cytotoxic effect	B6C3F1 mouse hepatocytes	
			F-344 rat hepatocytes	
	MTT assay DPPH assay	Cytotoxic activity Antioxidative properties	HL-60, HepG-2, WM266-4, SK-Mel-28, LCP-Mel, LCM-Mel, PNP-Mel, CN-MelA, and GR-Mel cells	[16, 48]
	WST assay SRB assay	Cytotoxic and genotoxic properties	V79 cells	[49]
	Corn oil gavage	Carcinogenic activity is based on increased incidences of hepatocellular adenoma, hepatocellular carcinoma, and hepatocellular adenoma or carcinoma (combined)	F344/N rats and B6C3F1 mice	[50]
	Trypan-blue exclusion assay	Cytotoxic activity	Rat hepatocytes	[55]

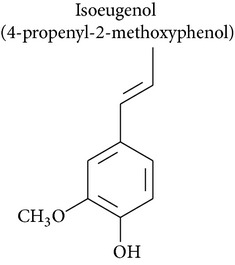	MTT assay	Cytotoxic activity	HSG cells	[29]
	DPPH radical-scavenging activity	Cormation of dimers		
	Dichlorofluorescein assay	Reduction in the intracellular level of GSH		[39]

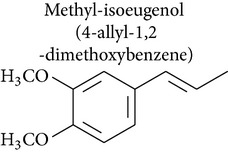	MTT assay	Inhibition of cell proliferation	WM266-4, SK-Mel-28, LCP-Mel, LCM-Mel, PNP-Mel, CN-MelA, and GR-Mel cells	[16]

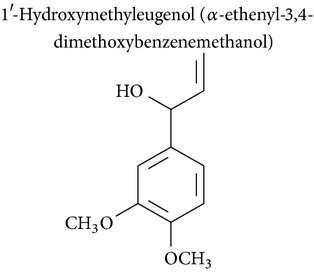	WST assay SRB assay	Cytotoxic and genotoxic properties	V79 cells	[49]

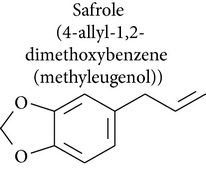	L-Lactate assay	Cytotoxic effect	B6C3F1 mouse hepatocytes	[47]
			F-344 rat hepatocytes	
	UDS assay	Cytotoxicity and genotoxicity effects	B6C3F1 mouse hepatocytes	
			F-344 rat hepatocytes	
	Trypan-blue exclusion assay	Potential cytotoxic effects	Rat hepatocytes and SCC-4 cells	[47, 51, 54]
	Flow cytometric assay	Induction of apoptosis of cells by involvement of mitochondria- and caspase-dependent signal pathway	SCC-4 cells	[51]
	Western blotting analysis	Upregulation of the protein expression of Bax and Bid and downregulation of the protein levels of Bcl-2 (upregulation of the ratio of Bax/Bcl-2), resulting in cytochrome c release, promoted Apaf-1 level, and sequential activation of caspase-9 and caspase-3 in a time-dependent manner		
	Real-time PCR	mRNA expressions of caspases 3, 8, and 9		
	MTT assay	Cytotoxic effect	Human BMFs	[52]
	Western blot analysis	Activate NF-*κ*B expression that may be involved in the pathogenesis of OSF and mediated by ERK activation and COX-2 signal transduction pathway		
	Fura-2 as a probe assay	Induced a [Ca^2+^]_*i*_ increase by causing Ca^2+^ release from the endoplasmic reticulum in a phospholipase C- and protein kinase C-independent fashion and by inducing Ca^2+^ influx	PC3 cells	[53]
	Comet assay/(DAPI) staining	Induced apoptosis (chromatin condensation) and DNA damage	HL-60 cells	[51]
	Flow cytometric analysis	Increased the production of reactive oxygen species (ROS) and Ca^2+^ and reduced the mitochondrial membrane potential		
	Western blotting analysis/confocal laser microscopy	Promoted the expression of glucose-regulated protein 78 (GRP78), growth arrest- and DNA damage-inducible gene 153 (GADD153), and activating transcription factor 6*α* (ATF-6*α*)		
	Flow cytometric analysis	Promoted the levels of CD11b and Mac-3 that might be the reason for promoting the activity of phagocytosis; reduced the cell population such as CD3 and CD19 cells	NK cells	[58]
	Ames test	Mutagenicity activity	*Salmonella* TA 98	[59]

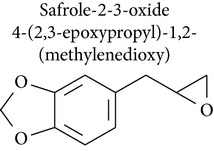	MTT assay	Produced toxicity in cells in a dose- and time-dependent manner	HepG2 cells FVB mice	[56]
	Comet assay	Significant dose-dependent increase in the degree of DNA (strand breaks)		
	Cytotoxic or genotoxic effect *in vivo*—i.p./Comet assay	Increase in mean Comet tail moment in peripheral blood leukocytes and in the frequency of micronucleated reticulocytes	HepG2 cells FVB mice	
	TUNEL assay	Activity of caspases 3, 8, and 9	A549 cells	[58]

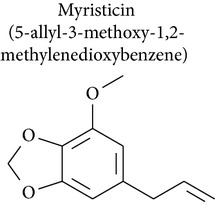	Western blot assay	Cleavages of PARP, accompanied by an accumulation of cytochrome c and by the activation of caspase-3	SK-N-SH cells	[60]

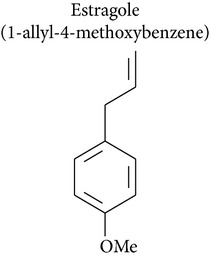	Induction of GST and QR	Induction of GST and QR in mouse livers	Four strains of mouse: A/JOlaHsd, C57BL/6NHsd, BALB/cAnNHsd, and CBA/JCrHsd	[61]
	Trypan-blue exclusion assay	Cytotoxic activity	Rat hepatocytes	[55]

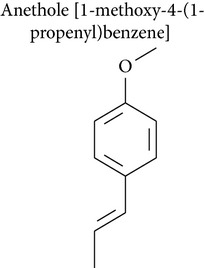	Trypan-blue assay	Cytotoxic activity	HeLa, rat hepatocytes cell	[21, 23, 55, 64]
	MTT assay	Cytotoxic activity	HT-1080, ML1-a cells	[63]
	Boyden-chamber assay	Reduced 40 and 85% of cells to invade into Matrigel	HT-1080 cells	[62]
	Gelatin zymography and RT-PCR analyses	Inhibitory effect of MMP-2 and MMP-9 and downregulate the expression of matrix metalloproteinases (MMPs) 2 and 9 and upregulate the gene expression of tissue inhibitor of metalloproteinase- (TIMP-) 1		
	Expression of MMPs, TIMPs, and uPA assays	Decreased mRNA expression of urokinase plasminogen activator (uPA)		
	Western blot analysis	Suppressed the phosphorylation of AKT, extracellular signal-regulated kinase (ERK), p38, and nuclear transcription factor kappa B (NF-*κ*B)		
	Fluorometric assay	Increases in the levels of ADP and AMP	Rat hepatocytes	[62]
	*CCK-8* assay	Estrogenic effect based on the concentrations of the hydroxylated intermediate, 4OHPB	MCF-7 cells	
	Western blot analysis	Suppressed TNF-induced activation of the transcription factor AP-1, c-jun N-terminal kinase, and MAPK-kinase	ML1-a cells	[63]
	Colorimetric e fluorometric assays	Reduced the levels of nucleic acids and MDA, and increased NP-SH concentrations	EAT cells in the paw of Swiss mice	[65]

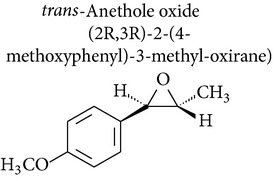	Ames test	Mutagenic for *Salmonella* tester strains	*Salmonella typhimurium* strains TA1535, TA100, and TA98	
	Induction of hepatic tumors	Carcinogenic in the induction of hepatomas	B6C3F1 mice	[67]
	Induction of skin papillomas	Carcinogenic in the induction of skin papillomas	CD-1 mice	

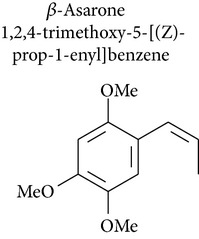	SRB assay	Cytotoxic activity	A549, SK-OV-3, SK-MEL-2, and HCT15 cells	[70]

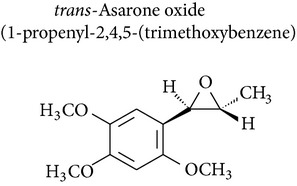	Ames test	Mutagenic for *Salmonella* tester strains	*Salmonella typhimurium* strains TA1535, TA100, and TA98	
	Induction of hepatic tumors	Carcinogenic in the induction of hepatomas	B6C3F1 mice	[67]
	Induction of skin papillomas	Carcinogenic in the induction of skin papillomas	CD-1 mice	

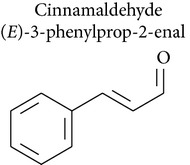	MTT assay	Cytotoxic activity	A375, HCT 116, MCF-7, P388, L-1210, 3LL, SNU-C5, HL-60, U-937, HCT 116, L1210 mouse, and Syrian hamster embryo cells	[71, 77, 78, 80, 84, 89]
	TRPA1 and TRPM8 gene expression	Reduce the proliferation of melanoma cells; this effect is independent of an activation of TRPA1 channels	A375, G361, SK-Mel-19, SK-Mel-23, and SK-Mel-28 cells	[77]
	Sulforhodamine B assay	Cytotoxic activity	HeLa, A549, SK-OV-3, SK-MEL-2, XF-498, and HCT-15 cells	[76]
	Ames test	Not mutagenic	Strains (TA 98, TA 100, TA 1535, and TA 1537) of *Salmonella typhimurium *	
	DTNB assay	TrxR inactivation	Recombinant rat TrxR	[78]
	Western blot analysis	Induced an adaptive antioxidant response through Nrf2-mediated upregulation of phase II enzymes, including TrxR induction	HCT 116 cells	
	XTT assay	Inhibitory effects on the growth of cells	Hep G2 cells	[80]
	Western blot analysis	Increase in the CD95 (APO-1/CD95) protein expression in Hep G2 cells		
		Inhibited the expression of Bax, p53, and CD95, as well as the cleavage of PARP. This pretreatment also prevented the downregulation of Bcl-XL in cells		
	Trypan-blue assay	Inhibited the proliferation of cells	PLC/PRF/5 cells	[81]
	Flow cytometer analysis	Activation of proapoptotic Bcl-2 family (Bax and Bid) proteins and MAPK pathway	PLC/PRF/5 cells	[83]
	Western immunoblot analysis	Prevented the phosphorylation of JNK and p38 proteins		
	DAPI/Fluorometric method	Induced apoptosis in cells	P388, L-1210, 3LL, SNU-C5, HL-60, U-937, and HepG2 cells	[71]
	Flow cytometry analysis	Induces the ROS-mediated mitochondrial permeability transition and resultant cytochrome c release		
	*cis*-DDP-induced	Potentiated the inactivating effect of *cis*-DDP in all phases of the cell cycle	NHIK 3025 cells	[82]
	NRU assay	Induced the fragmentation of nuclei (Plate 2), which is typical for condensed apoptotic phenotype	Hep-2 cells	[87]
	Genotoxicity assays—DNA repair test	Involve DNA damage as one of the factors involved in the mammalian cytotoxicity		
	LDH-cytotoxicity assay	Potent inhibitory effect against human hepatoma cell growth	HepG2 and Hep3B cells	[88]
	Western blot analysis	JAK2-STAT3/STAT5 pathway may be important targets		
		Decreased the protein levels of cyclin D1 and proliferative cell nuclear antigen (PCNA) but increased the protein levels of p27^Kip1^ and p21^Waf1/Cip1^		
	Flow cytometry analysis	Inducing apoptosis and synergizing the cytotoxicity of CIK cells	K562 cells	[92]
	Spectral analysis	Induced an adaptive antioxidant response through Nrf2-mediated upregulation of phase II enzymes, including TrxR induction	S180 in mice	[89]

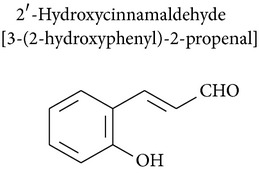	MTT assay	Cytotoxic activity	NIH/3T3 cells	[90]
	Lymphoproliferation—Con A, LPS, or PMA plus ionomycin	Inhibit the lymphoproliferation and induce a T-cell differentiation from CD4CD8 double positive cells to CD4 or CD8 single positive cells	Mice splenocytes	[74]
	Flow cytometry analysis	Capability to block the cell growth and stimulate a differentiation to mature cell		
	IgM-secreting B cells to SRBC	Decreased level of IgM to be due to the lower level of B-cell proliferation	Balb/c mice	

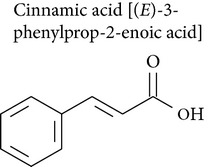	ELISA	Inhibits proliferation and DNA synthesis	Caco-2 cells	[79]
	Radioimmunoassay	Decreased intracellular cAMP levels		
	Flow cytometry analysis	Influence on the tumor cell cycle: G2-M period shortened, cell cycle lengthened, and cell proliferation inhibited	U14 cells	[92]
	*cis*-DDP-induced	Potentiated the inactivating effect of *cis*-DDP in all phases of the cell cycle	NHIK 3025 cells	[82]
	Trypan-blue assay	Anticancer activity	HL-60, A549, PC3, Du145, LN-CaP, A172, U251, SKMEL28, and A375 cells	[93, 94]
	Flow cytometry analysis	Inhibition and induced-differentiation on human osteogenic sarcoma cells	Human osteogenic sarcoma cells	[95]
	MTT assay	Cytotoxic activity	HepG2 cells	[97]
	Spectrophotometer	Higher antioxidant capacity		
	NRU assay	Cytotoxic activity	Mac Coy cells	[96]
	MTT assay	Antiviral activity	EHV-1	[98]

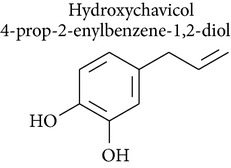	Trypan-blue assay	Cytotoxic activity	Rat hepatocytes	[54]
	Waters chromatograph	Decrease in cell viability, accompanied by losses of ATP, GSH; increase in GSSG, ROS, and MDA levels		

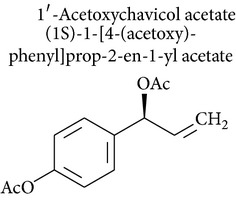	Indirect immunofluorescent method/EBV activation	Inhibiting the generation of anions during tumor promotion	Raji cells	[100]
	Trypan-blue exclusion assay	Cytotoxic activity	RPMI8226, U266, and IM-9 cells	[99]
	Flow cytometry	Induced caspases 3, 9, and 8 activities	RPMI8226 cells	
	Western blot analysis	TNF-*α*-induced apoptosis		
	ELISA	Downregulation of NF-*κ*B activity		
		TNF-*α*-induced apoptosis		
	*In vivo* assay	Anticancer effects with no toxic effects	NOD/SCID mouse	

## Abbreviations

### Cell Lines

3LL: Mouse lung carcinoma
A172: Human malignant glioblastoma
A375: Melanoma
A549: Lung adenocarcinoma
BMFs: Primary human buccal mucosal fibroblasts
Caco-2: Human colon adenocarcinoma
CD11b: Monocytes
CD19: B cells
CD3: T cells
CEM: Acute T lymphoblastoid leukemia
CN-Mel: Melanoma
DU-145: Androgen-insensitive prostate cancer
F344: Hepatocytes
G361: Melanoma
GR-Mel: Melanoma
HCT-15: Colon tumor
HeLa: Human cervical carcinoma
Hep3B: Human hepatoma cancer
HepG2: Human hepatoma
HGF: Human gingival fibroblasts
HL-60: Human promyelocytic leukemia
HSC-3: Human oral cancer cells
HSG: Human submandibular gland carcinoma
HT-1080: Human fibrosarcoma tumor
K-562: Human chronic myelogenous leukemia
KB: Oral squamous carcinoma
L-1210: Mouse leukemia
LCM-Mel: Melanoma
LCP-Mel: Melanoma
LN-CaP: Prostate cancer
Mac-3: Macrophages
MCF-7 gem: Human breast adenocarcinoma (resistant to gemcitabine)
MCF-7: Human breast adenocarcinoma
ML-1: Human myeloblastic leukemia
NHIK 3025: Human cervical carcinoma
P388: Mouse leukemia
P-815: Murine mastocytoma
PC-3: Human prostate cancer
PLC/PRF/5: Human hepatoma
PNP-Mel: Melanoma
Raw 264.7: Mouse leukemic monocyte macrophage
S180: Sarcoma 180
SbCl2: Primary melanoma
SCC-4: Tongue squamous carcinoma
SK-Mel-19: Melanoma
SK-MEL-2: Skin melanoma
SK-MEL-23: Melanoma
SK-MEL-28: Melanoma
SK-N-SH: Neuroblastoma
SK-OV-3: Ovarian cancer
SNU-C5: Human colon cancer
U14: Uterocervical carcinoma
U251: Human malignant glioblastoma
U-937: Human histiocytic lymphoma
uPA: Urokinase plasminogen activator
WM1205Lu: Metastatic melanoma
WM266-4: Melanoma
WM3211: Primary radial growth phase melanoma
WM98-1: Primary vertical growth phase melanoma
XF-498: Central nerve system.

### Tests

AFC: Antibody forming cell
ALDH: Aldehyde dehydrogenase
Ames test: Biological assay to assess the mutagenic potential of chemical compounds
Boyden-chamber assay: Evaluation of tumor cell invasion* in vitro*
c-AMP: Cyclic adenosine monophosphate
CAs: Chromosomal aberrations
CCK-8: Cell Counting Kit-8, a sensitive colorimetric assay
CDFHDA: 5-(and -6)-carboxy-2′,7′-dichlorofluorescein diacetate
Comet assay: Single-cell gel electrophoresis
Con A: Concanavalin
DAPI: 4′,6-Diamidino-2-phenylindole
DCFH: Dichlorofluorescein
DEM: Diethyl maleate
DMBA: 7,12-Dimethylbenz[a]anthracene
DPPH: 1,1-Diphenyl-2-picrylhydrazyl
DTNB: 5,5′-Dithiobis-(2-nitrobenzoic acid)
EBV: Epstein-Barr virus
EHV-1: Herpes virus 1
ESR: Electron spin resonance spectroscopy
GSSG: Oxidized glutathione
GST: Glutathione S-transferase
LDH: Lactate dehydrogenase
LPS: Lipopolysaccharide
MDA: Malondialdehyde
MMP: Matrix metalloproteinase
MTT: [3(4,5-Dimethyl-thiazol-2-yl)-2,5-diphenyl tetrazolium bromide]
NF-*κ*B: Nuclear factor-kappa B
NRU assay: Neutral red uptake
p21WAF1: Cyclin-dependent kinase inhibitor CDKN1A
PARP: Poly(ADP-ribose) polymerase
PBS: Phosphate-buffered saline
PCR: Polymerase chain reaction
PMA: Phorbol 12-myristate-13-acetate plus ionomycin
QR: Quinone oxidoreductase
SRB: Sulforhodamine B
SRBC: Sheep red blood cells
TBA: Test in the aqueous phase
TBARS: Thiobarbituric acid reactive substances
TUNEL: Terminal deoxynucleotidyl transferase-mediated dUTP nick end-labeling
UDS assay: Unscheduled DNA synthesis
V-FITC assay: Apoptosis detection kit
WST: Tetrazolium salt
XTT: 2,3-Bis-(2-methoxy-4-nitro-5-sulfophenyl)-2H-tetrazolium-5-carboxanilide.
